# Self-medication among university students in Guangdong province, China: a cross-sectional study using the KAP model

**DOI:** 10.3389/fpubh.2025.1601731

**Published:** 2025-07-14

**Authors:** Lu Tan, Yuting Chen, Meiling Gu, Jiangwei Zhu, Xiaoyan Tang, Wenying Chen, Huancun Feng

**Affiliations:** ^1^Department of Pharmacy, The Third Affiliated Hospital, Southern Medical University, Guangzhou, China; ^2^Department of Pharmacy, Guangzhou Red Cross Hospital of Jinan University, Guangzhou, Guangdong Province, China; ^3^Pharmacy Center, Guangzhou Geriatric Hospital, Guangzhou, Guangdong Province, China

**Keywords:** university students, self-medication, KAP model, rational medication use, Guangdong province

## Abstract

**Objective:**

Self-medication has emerged as a significant global public health concern. Despite possessing a certain level of medication knowledge, university students in China exhibit a high-risk profile regarding self-medication practices. This study aimed to systematically investigate the current status and influencing factors of self-medication among university students in Guangdong Province, China, thereby providing evidence-based recommendations for targeted intervention strategies.

**Methods:**

A cross-sectional study was conducted based on the Knowledge-Attitude-Practice (KAP) model. Data were collected via anonymous questionnaire surveys distributed to university students in Guangdong Province, China. A total of 816 valid responses were analyzed. The questionnaire assessed demographic characteristics along with dimensions of medication knowledge, attitudes, and practices. Multiple linear regression analyses were subsequently performed to evaluate the impact of demographic factors on each dimension of the KAP model.

**Results:**

Students demonstrated a relatively high overall qualification rate in medication knowledge (93.50%), with 43.38% achieving a “Good” level and 50.12% rated as “Fair.” However, noticeable deficiencies were identified in attitudes and practices, with qualification rates approximately 75% in both dimensions. Notably, only 6.50% achieved a “Good” level in medication practices, while a substantial proportion (24.26%) was rated as “Unqualified.” Regression analyses revealed that age, current academic stage, and study mode significantly influenced medication knowledge scores. No significant demographic factors were associated with medication attitudes. However, age and the primary source of medication information significantly impacted self-medication practices. These findings offer empirical evidence essential for developing targeted medication safety education interventions among university students.

**Conclusion:**

A clear discrepancy between knowledge and practice regarding self-medication exists among university students in Guangdong Province, China. Comprehensive intervention strategies are therefore, urgently required to promote rational medication behaviors within this population.

## 1 Background

According to the World Health Organization (WHO), self-medication is defined as “the selection and use of medicines by individuals to treat self-recognized illnesses or symptoms, or the intermittent or continued use of a medication prescribed by a physician for chronic or recurrent diseases or symptoms” (1). In recent years, driven by the increasing strain on global healthcare resources and heightened public health awareness, self-medication practices have become increasingly prevalent worldwide (2). A systematic review indicates that the global average prevalence of self-medication is approximately 67%, with notable variations across regions: Eastern Europe (74%), Asia (71.2%), South America (60.0%), and Africa (55.9%) (3). At the country level, self-medication rates range from 41.5 to 75.5% in Asian nations like India, Bhutan, Bangladesh, and Saudi Arabia (4–6); 53.7% in Ghana, Africa (7); and typically exceed 50% in several European countries including Finland, Lithuania, Cyprus, Denmark, and Hungary (8). These variations are often closely linked to healthcare accessibility, public health literacy, economic development levels, and cultural attitudes toward medication use.

Although self-medication can enhance individuals' capacity for managing minor ailments independently, its associated risks require serious consideration. Insufficient medical knowledge or cognitive biases may lead to inappropriate or irrational medication use, resulting in delayed diagnosis, increased antimicrobial resistance, adverse drug reactions, and, in severe cases, life-threatening consequences (9, 10).

Currently, inappropriate medication use represents a major global public health challenge requiring urgent action (11). In response, WHO launched the “Medication Without Harm” Global Patient Safety Challenge in 2017, aiming to reduce severe avoidable medication-related harm globally by 50% within 5 years. This initiative emphasizes the crucial role of public education in promoting rational medicine use, aiming to enhance awareness of medication safety and thereby mitigate health risks associated with medication misuse and abuse (12, 13).

In China, despite substantial improvements in primary healthcare coverage, high-quality medical resources remain concentrated in urban areas, leaving rural populations underserved (14, 15). This disparity indirectly contributes to the high prevalence of self-medication. Existing research indicates that self-medication is notably common among the Chinese population. A large nationwide cohort study reported an exceptionally high rate of 99.1%; however, this figure is likely inflated due to broad inclusion criteria encompassing any instance of self-administered over-the-counter (OTC) medication use (16).

Within this context, irrational medication use among university students warrants special attention. Although university students typically possess higher educational attainment and advanced information-seeking abilities, their actual medication behaviors often lack corresponding rationality (17). Studies focusing on antibiotic self-medication among Chinese university students have identified concerning trends. For example, a survey conducted in Shanxi Province reported that 40.2% of students had self-medicated with antibiotics within the previous 6 months, 59.2% of whom obtained antibiotics without prescriptions, and exhibited generally low levels of antibiotic-related knowledge (18). Similarly, a study from Jiangsu Province found that 47.9% of students self-medicated with antibiotics, holding misconceptions such as the belief that antibiotics are effective against viral infections (19). These findings collectively highlight substantial knowledge gaps and prevalent inappropriate medication practices among university students.

China hosts one of the world's largest higher education systems, with Guangdong Province alone comprising 154 universities and colleges and enrolling over 2.6 million students (20, 21). Despite generally strong access to information, university students remain particularly susceptible to self-medication risks due to unique factors including inconsistent decision-making, peer influence, and chronic academic stress (11, 22–24). Studies further indicate that students from less developed regions may be more inclined to purchase and use antibiotics independently (25, 26), and cultural differences could further influence their medication behaviors (27). University students' medication practices affect not only individual health but also have broader societal implications through peer influence and intergenerational patterns. Thus, medication safety among university students constitutes a complex public health issue necessitating interdisciplinary collaboration and targeted governance strategies.

To comprehensively analyze university students' medication behaviors, the Knowledge-Attitude-Practice (KAP) model provides an effective theoretical framework. Recognized for its simplicity, quantifiable indicators, and interpretability, the KAP model has become a standard tool in evaluating healthcare services (28, 29). This model describes the formation of health behaviors as a sequential continuum involving knowledge acquisition, attitude development, and behavioral implementation. Within this framework, medication knowledge forms the cognitive foundation, attitudes serve as mediators, and practices represent behavioral outcomes (30). Although various studies have examined medication practices among the general Chinese population, medical students, and healthcare professionals (31–35), specific and systematic investigations targeting university students in Guangdong Province remain limited. Therefore, this study utilizes the KAP model to investigate medication safety among university students in Guangdong Province, aiming to provide theoretical support for developing targeted educational strategies within higher education institutions.

## 2 Methods

### 2.1 Study design and participants

This cross-sectional study was conducted via anonymous online surveys using Wenjuanxing, a widely-used survey platform in China, among university students in Guangdong Province from February 1 to March 15, 2025. The study protocol was approved by the Institutional Review Board of the Third Affiliated Hospital of Southern Medical University (Approval no.: 2025-ER-008) and strictly adhered to the principles of the Declaration of Helsinki. Electronic informed consent was voluntarily provided by all participants prior to participation in the study. Given the non-sensitive nature of the survey content and absence of complex legal implications, according to national legislation and institutional requirements, university students aged 16–17 years were deemed competent to provide informed consent independently; therefore, additional written informed consent from their legal guardians or next of kin was not required.

#### 2.1.1 Eligibility criteria

*Inclusion criteria were as follows:*

1. Currently enrolled in Guangdong higher education institutions (including college diploma students, undergraduate students, master's students, and doctoral students).
2. Aged ≥16 years.
3. Voluntarily provided electronic informed consent.

*Exclusion criteria were as follows:*

1. Questionnaire completion time < 3 min.
2. Missing data for key variables.
3. Presence of logical contradictions or illogical response patterns. An “illogical response pattern” was defined as responses exhibiting distinct patterns, extremity bias, or mechanical repetition across a large block of consecutive questions, or providing answers that clearly contradict common sense on blatantly obvious questions (e.g., selecting “Strongly Agree” for the statement: “Storing medications where children can easily access them”).

Questionnaires fulfilling any of these criteria were identified and excluded via manual review by the research team to ensure data quality and reliability.

#### 2.1.2 Ethical compliance and quality assurance

1. The first page of the questionnaire clearly stated the research purpose, guaranteed anonymity, and informed participants of their right to withdraw. Participation required active selection of the “Agree” option to proceed.
2. To prevent duplicate submissions and malicious responses, an IP address restriction was implemented (allowing only one submission per IP address), and questionnaire completion time was validated.
3. Following data export, two researchers independently reviewed the data to ensure quality. Discrepancies were resolved by consensus between the researchers regarding any necessary exclusions.

### 2.2 Questionnaire development and validity testing

The questionnaire structure was developed based on the framework of the “Residents' Medication Behavior Risk KAP Questionnaire” from the Chinese Pharmaceutical Association (33), which underwent revisions through the Delphi expert consultation method with three pharmaceutical professors to achieve localized adaptation for university students' medication scenarios (see Supplementary Material 1 for details). The finalized questionnaire comprised two sections: demographic characteristics (10 items), serving as independent variables for subsequent analysis; and KAP dimensions, including medication knowledge (28 items), medication attitudes (13 items), and medication practices (25 items).

#### 2.2.1 Standardized scoring criteria

All Likert-scale items were positively scored (ranging from one = “Strongly Disagree/Never” to five = “Strongly Agree/Always”). Based on previous studies (33, 35), scores were categorized as follows:

*Knowledge dimension*: scores of 28–56 (Good), 57–84 (Fair), and ≥85 (Unqualified). Lower scores indicate better rational medication knowledge.
*Attitude dimension*: scores of 52–65 (Good), 39–51 (Fair), and ≤ 38 (Unqualified). Higher scores indicate more cautious and rational medication attitudes.
*Practice dimension*: scores of 25–50 (Good), 51–75 (Fair), and ≥76 (Unqualified). Lower scores indicate more proactive medication practices.

#### 2.2.2 Validity and reliability testing

Following revision, the questionnaire was pre-tested with a random sample of 50 university students; data from the pre-test were excluded from the formal analysis. The overall Cronbach's alpha coefficient of the questionnaire was 0.964, indicating excellent internal consistency reliability. The dimension-specific Cronbach's alpha values were 0.972 (Knowledge), 0.939 (Attitude), and 0.917 (Practice), demonstrating high reliability (α > 0.8). For construct validity, the Kaiser-Meyer-Olkin (KMO) measure of sampling adequacy was 0.961 overall, with values of 0.968 (Knowledge), 0.934 (Attitude), and 0.936 (Practice), exceeding the recommended standard (KMO >0.8). Bartlett's test of sphericity was significant (*p* < 0.001), confirming good structural validity and suitability of the questionnaire for this study.

### 2.3 Sample size calculation

Based on data released by the Guangdong Provincial Department of Education (21), the total college student population in Guangdong Province is approximately 2.6 million. The required sample size was calculated using the following formula:


$$\begin{array}{l} n=\frac{Z^{2}\times p\times \left(1-p\right)}{E^{2}} \end{array}$$

where *Z* = 1.96 (95% confidence level), *p* = 0.5, and *E* = 0.05. This yielded a minimum sample size of 385. Accounting for a 10% invalid responses and a design effect (*Deff* = 1.2), the adjusted sample size was: *n*′ = 385 × (1 + 0.1) × 1.2 = 512.

### 2.4 Statistical analysis

Statistical analysis was performed using SPSS software (version 26.0; IBM Corp., Armonk, NY, USA). Demographic characteristics were described using frequencies and percentages [*n* (%)]. As the KAP scores were not normally distributed, they were described using median and interquartile range (IQR). Inter-group comparisons of KAP scores were performed using the Mann–Whitney *U*-test (for two groups) or the Kruskal–Wallis *H*-test (for three or more groups), based on the number of groups. To further explore the factors influencing scores in each dimension of the KAP, multiple linear regression models were constructed with the scores of each dimension as the dependent variable. All variables significant in the univariate analyses were included in these models. The unstandardized regression coefficients (*B*) and standardized regression coefficients (β) were calculated. Multicollinearity within the models was assessed using the variance inflation factor (VIF) and tolerance. *p*-value < 0.05 was considered statistically significant for all analyses.

## 3 Results

### 3.1 Characteristics of study participants and univariate analysis of demographic variables

A cross-sectional survey design was employed in this study. A total of 880 questionnaires were distributed. After excluding invalid responses, 816 valid questionnaires remained, yielding a valid response rate of 92.7%. The sample comprised 430 males (52.7%) and 386 females (47.3%). The predominant age group was 18–20 years (70.2%). Most respondents were enrolled in diploma programs (71.1%) or undergraduate programs (23.4%). The majority were non-medical/non-pharmaceutical majors (72.5%), enrolled in full-time programs (95.5%), and attended vocational colleges (66.5%). Participants' urban and rural residences were nearly evenly distributed (urban: 50.9%; rural: 49.1%). The most frequently reported monthly household income per capita was 3,000 to 5,000 RMB, accounting for 29.5% of respondents.

Table 1 presents the analysis of relationships between demographic variables and KAP dimension scores. The results indicated that gender showed no statistically significant differences across any KAP dimensions (*p* > 0.05). However, significant differences (*p* < 0.05) emerged across various dimensions for age, current education level, major, study mode, institution type, institution region, urban/rural residence, family income, and sources of medication knowledge in some dimensions.

**Table 1:** Demographic characteristics and factors associated with medication-related KAP scores among students (*n* = 816).

**Variables**	**Knowledge score**	**Statistic**	***p*-Value**	**Attitude score**	**Statistic**	***p*-Value**	**Practice score**	**Statistic**	***p*-Value**
**Gender**									
Male (*n* = 430)	58 (47, 73.75)	82,155	0.470	39 (32, 43)	80,542	0.465	63 (56, 75)	84,447	0.664
Female (*n* = 386)	58 (48.25, 66)			38 (34, 42)			62 (58, 69)		
**Age**									
16–17 岁 (*n* = 11)	64 (42.5, 91.5)	15.506	**< 0.001**	39 (34.5, 43.5)	2.353	0.671	74 (63.5, 79.5)	9.745	**0.045**
18–20 岁 (*n* = 573)	58 (49, 70)			38 (32, 42)			62 (57, 72)		
21–24岁 (*n* = 191)	58 (49, 67)			38 (33, 41)			64 (59, 71.5)		
25–28 岁 (*n* = 24)	41.5 (33.75, 53.25)			39 (37, 45)			62.5 (57, 66.25)		
Over 28 岁 (*n* = 17)	56 (51, 82)			39 (36, 46)			67 (60, 83)		
**Current academic stage**									
College diploma student (*n* = 580)	59 (50, 73)	12.585	**< 0.001**	38 (32, 43)	1.578	0.664	62 (57, 74.25)	9.910	**0.019**
Undergraduate student (*n* = 191)	57 (45, 66)			38 (33, 41)			64 (60, 71)		
Master's student (*n* = 35)	51 (34, 60)			38 (36, 45)			64 (59, 67.5)		
Doctoral student (*n* = 10)	39.5 (38, 52.75)			38.5 (33.25, 42.25)			56.5 (49.75, 62.75)		
**Major**									
Medical/pharmaceutical-related (*n* = 224)	57 (46, 65)	58,444.5	**0.009**	39 (33.75, 43.25)	69,977.5	0.220	61 (57, 68.25)	60,016	**0.036**
Non-medical/pharmaceutical (*n* = 592)	58 (49, 71)			38 (32, 41)			63 (57, 74)		
**Study mode**									
Full-time (*n* = 779)	58 (47, 68)	10,294.5	**0.009**	38 (33, 42)	12,908	0.282	62 (57, 72)	11,323	**0.027**
Part-time (*n* = 37)	63 (52, 84)			39 (33, 52)			68 (60, 81)		
**School type**									
Double first-class university (*n* = 65)	58 (43, 69)	9.274	**0.001**	38 (34, 41)	0.314	0.855	66 (59, 75)	5.305	0.070
Non-double first-class university (*n* = 208)	56 (41.75, 64.25)			38 (33, 43)			64 (59, 71)		
Vocational college (*n* = 543)	59 (50, 72.5)			38 (32, 42)			62 (57, 74)		
**Location of your school**									
Central urban area (*n* = 283)	57 (46, 71.5)	73,665	0.534	39 (33, 44)	81,375.5	0.062	64 (57, 75)	81,987.5	0.040
Non-central urban area (*n* = 533)	58 (49, 68)			38 (32, 41)			62 (57, 71)		
**Family residence**									
Rural (*n* = 401)	59 (50, 70)	85,953.05	**0.024**	38 (32, 41)	77,574	0.093	62 (57, 72)	83,193.5	0.997
Urban (*n* = 415)	57 (45, 67)			39 (33, 44)			63 (57, 72)		
**Monthly household income per capita (CNY)**									
Below ¥1,000 (*n* = 56)	63.5 (43, 80)	4.693	0.676	38 (32, 44.25)	10.078	**0.039**	62 (57, 75)	4.298	0.367
¥1,000–3,000 (*n* = 167)	59 (49.5, 67)			38 (32, 39)			63 (58.5, 71)		
¥3,000–5,000 (*n* = 241)	58 (48, 67)			39 (34, 43)			62 (57, 71)		
¥5,000–8,000 (*n* = 193)	57 (46, 66)			38 (32, 42)			62 (56, 69)		
Above ¥8,000 (*n* = 159)	58 (47, 75)			39 (33, 44)			65 (58, 75)		
**Primary source of medication knowledge**									
Family (*n* = 254)	58 (50, 74)	8.88	0.009	38 (32, 41)	4.169	0.384	63 (58, 75)	5.987	0.200
Internet (*n* = 312)	59 (49, 68.25)			38.5 (33, 41)			62.5 (57, 71)		
School (*n* = 152)	56 (46.75, 64.25)			39 (35, 44)			62 (57, 69.25)		
Friends/peers (*n* = 17)	48 (38, 58)			38 (31, 40)			54 (37, 65)		
Books, newspapers, magazines (*n* = 81)	58 (47, 69)			39 (33, 43)			63 (57, 74)		

Values in bold indicate statistical significance (*P* < 0.05).

### 3.2 Current status of KAP scores regarding self-medication among university students

The KAP scores of the respondents concerning self-medication are presented in Table 2. Overall, students showed the highest qualification rate in the knowledge dimension (93.50%). Within this dimension, 43.38% achieved a “Good” level, and 50.12% were at a “Fair” level.

**Table 2:** Qualification rates of KAP scores among participants.

**Category**	**Median (IQR)**	**Unqualified, *n* (%)**	**Fair, *n* (%)**	**Good, *n* (%)**	**Qualification rate (%)**
Knowledge score	58 (48, 69)	53 (6.50%)	409 (50.12%)	354 (43.38%)	93.50%
Attitude score	38 (33, 42)	202 (24.75%)	426 (52.21%)	188 (23.04%)	75.25%
Practice score	63 (57, 72)	198 (24.26%)	565 (69.24%)	53 (6.50%)	75.74%

The qualification rate in the attitude dimension was 75.25%, with 23.04% achieving a “Good” level and 52.21% at a “Fair” level. However, 24.75% of students failed to meet the qualification criteria in this dimension.

Regarding the practice dimension, while the qualification rate (75.74%) was comparable to that of the attitude dimension, the proportion achieving a “Good” level was significantly lower (only 6.50%). Students at a “Fair” level accounted for 69.24%, and the proportion of students classified as “Unqualified” was 24.26%.

### 3.3 Multivariable linear regression analysis of demographic factors on self-medication KAP scores

Multiple linear regression models were used to analyze the influence of demographic variables on self-medication KAP scores. The detailed results are presented separately for knowledge, attitude, and practice scores.

As shown in Table 3, compared with students aged 16–17 years, significantly lower knowledge scores were observed in students aged 18–20 years (*B* = −14.990, 95% CI: −20.475 to −7.833, *p* = 0.018), 21–24-year-old (*B* = −12.825, 95% CI: −17.579 to −4.633, *p* = 0.048), and 25–28-year-old (*B* = −23.370, 95% CI: −28.324 to −11.946, *p* = 0.004). Undergraduate students (*B* = −7.589, 95% CI: −10.062 to −3.002, *p* = 0.032) and master's degree students (*B* = −11.076, 95% CI: −14.902 to −3.668, *p* = 0.049) demonstrated significantly lower knowledge scores than diploma students. Part-time students had significantly higher knowledge scores than full-time students (*B* = 12.100, 95% CI: 9.110–16.378, *p* = 0.001). Furthermore, students primarily obtaining medication knowledge from friends or peers had significantly lower scores than those whose primary source was family (*B* = −14.453, 95% CI: −17.176 to −6.878, *p* = 0.005).

**Table 3:** Multivariate linear regression analysis of demographic factors associated with knowledge scores regarding self-medication.

**Variables**	**Estimate (*B*)**	**Std. estimate (β)**	**Std. error (SE)**	**95% CI**	***t***	***p*-Value**	**VIF**	**Tolerance**
**Gender**								
Male	Ref						1.123	0.889
Female	0.052	0.034	1.511	(−0.431, 2.591)	0.034	0.973		
**Age**								
16–17	Ref						3.058	0.325
18–20	−14.990	−2.371	6.321	(−20.475, −7.833)	−2.371	0.018		
21–24	−12.825	−1.982	6.472	(−17.579, −4.633)	−1.982	0.048		
25–28	−23.370	−2.854	8.189	(−28.324, −11.946)	−2.854	0.004		
Over 28	−10.461	−1.244	8.411	(−14.769, 2.065)	−1.244	0.214		
**Current academic stage**								
College diploma student	Ref						10.202	0.098
Undergraduate student	−7.589	−2.151	3.528	(−10.062, −3.002)	−2.151	0.032		
Master's student	−11.076	−1.971	5.619	(−14.902, −3.668)	−1.971	0.049		
Doctoral student	−15.932	−1.945	8.189	(−20.831, −4.457)	−1.945	0.052		
**Major**								
Medical/pharmaceutical-related	Ref						1.515	0.656
Non-medical/pharmaceutical	0.988	0.503	1.963	(−0.132, 3.804)	0.503	0.615		
**Study mode**								
Full-time	Ref						1.125	0.885
Part-time	12.100	3.334	3.629	(9.11, 16.378)	3.334	0.001		
**School type**								
Double first-class university	Ref						5.283	0.187
Non-double first-class university	−3.283	−1.061	3.095	(−4.998, 1.206)	−1.061	0.289		
Vocational college	−3.347	−0.855	3.915	(−4.943, 2.901)	−0.855	0.393		
**Location of your school**								
Central urban area	Ref						1.197	0.833
Non-central urban area	−2.965	−1.812	1.636	(−3.455, −0.179)	−1.812	0.070		
**Family residence**								
Rural	Ref						1.196	0.835
Urban	−1.870	−1.201	1.557	(−2.432, 0.684)	−1.201	0.230		
**Monthly household income per capita (CNY)**								
Below ¥1,000	Ref						1.295	0.763
¥1,000–3,000	−4.268	−1.344	3.176	(−6.242, 0.116)	−1.344	0.179		
¥3,000–5,000	−3.555	−1.149	3.094	(−4.366, 1.83)	−1.149	0.251		
¥5,000–8,000	−2.748	−0.853	3.222	(−2.662, 3.79)	−0.853	0.394		
Above ¥8,000	−2.325	−0.702	3.312	(−1.284, 5.35)	−0.702	0.483		
**Primary source of medication knowledge**								
Family	Ref						1.444	0.692
Internet	−1.480	−0.847	1.747	(−2.062, 1.436)	−0.847	0.397		
School	−2.142	−0.897	2.387	(−2.308, 2.472)	−0.897	0.370		
Friends/peers	−14.453	−2.812	5.140	(−17.176, −6.878)	−2.812	0.005		
Books, newspapers, magazines	−0.596	−0.223	2.671	(0.918, 6.268)	−0.223	0.824		
*R* ^2^	0.082							
Adjusted *R*^2^	0.057							
*F*	*F* (22, 793) = 3.24, *p* = 0.000							

As indicated in Table 4, no significant associations were identified between demographic variables and attitude scores.

**Table 4:** Multivariate linear regression analysis of demographic factors associated with attitude scores regarding self-medication.

**Variables**	**Estimate (*B*)**	**Std. estimate (β)**	**Std. error (SE)**	**95% CI**	***t***	***p*-Value**	**VIF**	**Tolerance**
**Gender**								
Male	Ref						1.125	0.889
Female	0.653	0.858	0.760	−0.837, 2.143	0.858	0.391		
**Age**								
16–17	Ref						3.079	0.325
18–20	−2.199	−0.691	3.180	−8.432, 4.034	−0.691	0.490		
21–24	−2.389	−0.733	3.256	−8.771, 3.993	−0.733	0.463		
25–28	−3.216	−0.781	4.120	−11.291, 4.859	−0.781	0.435		
Over 28	−3.742	−0.884	4.234	−12.041, 4.556	−0.884	0.377		
**Current academic stage**								
College diploma student	Ref						10.253	0.098
Undergraduate student	−0.836	−0.471	1.776	−4.317, 2.645	−0.471	0.638		
Master's student	0.898	0.318	2.826	−4.641, 6.437	0.318	0.751		
Doctoral student	−2.530	−0.614	4.119	−10.603, 5.543	−0.614	0.539		
**Major**								
Medical/pharmaceutical-related	Ref						1.524	0.656
Non-medical/pharmaceutical	0.287	0.290	0.990	−1.653, 2.227	0.290	0.772		
**Study mode**								
Full-time	Ref						1.129	0.885
Part-time	2.994	1.638	1.828	−0.589, 6.579	1.638	0.102		
**School type**								
Double first-class university	Ref						5.343	0.187
Non-double first-class university	1.543	0.989	1.561	−1.517, 4.603	0.989	0.323		
Vocational college	1.099	0.557	1.973	−2.768, 4.966	0.557	0.578		
**Location of your school**								
Central urban area	Ref						1.201	0.833
Non-central urban area	−0.959	−1.164	0.824	−2.5746, 0.656	−1.164	0.245		
**Family residence**								
Rural	Ref						1.198	0.835
Urban	1.034	1.320	0.784	−0.503, 2.571	1.320	0.187		
**Monthly household income per capita (CNY)**								
Below ¥1,000	Ref						1.310	0.763
¥1,000–3,000	0.341	0.213	1.599	−2.793, 3.475	0.213	0.831		
¥3,000–5,000	2.513	1.612	1.558	−0.541, 5.567	1.612	0.107		
¥5,000–8,000	1.170	0.721	1.623	−2.011, 4.351	0.721	0.471		
Above ¥8,000	2.235	1.339	1.669	−1.036, 5.506	1.339	0.181		
**Primary source of medication knowledge**								
Family	Ref						1.465	0.683
Internet	0.014	0.016	0.88	−1.711, 1.739	0.016	0.987		
School	0.415	0.345	1.202	−1.941, 2.771	0.345	0.730		
Friends/peers	−1.238	−0.478	2.590	−6.314, 3.838	−0.478	0.633		
Books, newspapers, magazines	0.672	0.499	1.346	−1.966, 3.310	0.499	0.618		
*R* ^2^	0.024							
Adjusted *R*^2^	0.003							
*F*	*F* (22, 793) = 0.878, *p* = 0.626							

As presented in Table 5, practice scores were significantly lower in the 18–20-year-old (*B* = −12.973, 95% CI: −22.042 to −3.904, *p* = 0.005) and 21–24-year-old (*B* = −10.980, 95% CI: −20.266 to −1.694, *p* = 0.021) groups compared with the 16–17-year-old group. Moreover, part-time students demonstrated significantly higher practice scores compared to full-time students (*B* = 7.102, 95% CI: 1.888–12.316, *p* = 0.008), while those citing friends or peers as their primary source had significantly lower practice scores (*B* = −11.522, 95% CI: −18.909 to −4.135, *p* = 0.002).

**Table 5:** Multivariate linear regression analysis of demographic factors associated with practice scores regarding self-medication.

**Variables**	**Estimate (*B*)**	**Std. estimate (β)**	**Std. error (SE)**	**95% CI**	***t***	***p*-Value**	**VIF**	**Tolerance**
**Gender**								
Male	Ref						1.125	0.889
Female	−0.319	−0.288	1.106	−2.487, 1.849	−0.288	0.773		
**Age**								
16–17	Ref						3.079	0.325
18–20	−12.973	−2.804	4.627	−22.042, −3.904	−2.804	0.005		
21–24	−10.980	−2.317	4.738	−20.266, −1.694	−2.317	0.021		
25–28	−11.510	−1.920	5.994	−23.258, 0.238	−1.920	0.055		
Over 28	−7.334	−1.190	6.161	−19.410, 4.742	−1.190	0.234		
**Current academic stage**								
College diploma student	Ref						10.253	0.098
Undergraduate student	1.282	0.496	2.584	−3.782, 6.347	0.496	0.620		
Master's student	−0.549	−0.133	4.112	−8.609, 7.511	−0.133	0.894		
Doctoral student	−12.384	−2.067	5.993	−24.130, −0.638	−2.067	0.039		
**Major**								
Medical/pharmaceutical-related	Ref						1.524	0.656
Non-medical/pharmaceutical	1.157	0.803	1.441	−1.667, 3.981	0.803	0.422		
**Study mode**								
Full-time	Ref						1.129	0.885
Part-time	7.102	2.670	2.660	1.888, 12.316	2.670	0.008		
**School type**								
Double first-class university	Ref						5.343	0.187
Non-double first-class university	−2.445	−1.077	2.271	−6.896, 2.006	−1.077	0.282		
Vocational college	−1.177	−0.410	2.871	−6.804, 4.450	−0.410	0.682		
**Location of your school**								
Central urban area	Ref						1.201	0.833
Non-central urban area	−0.708	−0.590	1.199	−3.058, 1.6420	−0.590	0.555		
**Family residence**								
Rural	Ref						1.198	0.835
Urban	0.249	0.219	1.140	−1.985, 2.483	0.219	0.827		
**Monthly household income per capita (CNY)**								
Below ¥1,000	Ref						1.31	0.763
¥1,000–3,000	−1.11	−0.477	2.327	−5.671, 3.451	−0.477	0.633		
¥3,000–5,000	0.021	0.009	2.267	−4.422, 4.464	0.009	0.993		
¥5,000–8,000	−0.768	−0.325	2.361	−5.396, 3.860	−0.325	0.745		
Above ¥8,000	1.210	0.498	2.428	−3.549, 5.969	0.498	0.618		
**Primary source of medication knowledge**								
Family	Ref						1.465	0.683
Internet	−0.840	−0.656	1.280	−3.349, 1.669	−0.656	0.512		
School	−0.324	−0.185	1.749	−3.752, 3.104	−0.185	0.853		
Friends/peers	−11.522	−3.057	3.769	−18.909, −4.135	−3.057	0.002		
Books, newspapers, magazines	0.704	0.359	1.958	−3.134, 4.542	0.359	0.719		
*R* ^2^	0.053							
Adjusted *R*^2^	0.021							
*F*	*F* (22, 793) = 2.019, *p* = 0.003							

The regression models accounted for limited variance, with adjusted *R*^2^ values of 0.057 for the knowledge score model and 0.021 for the practice score model, suggesting that additional influential factors not included in these models might exist.

## 4 Discussion

### 4.1 Comparison of main findings with previous literature

The results of this study indicate the presence of a significant gap between KAP regarding self-medication among the university student population. Although participants demonstrated overall high levels of medication knowledge, notable deficiencies were observed in their medication-related attitudes, with a considerable proportion holding negative or ambiguous perceptions toward safe medication practices. Issues related to actual medication behaviors were even more pronounced. Students who possessed adequate knowledge frequently failed to implement safe medication practices effectively.

Comparisons with previous studies reveal both similarities and differences. A KAP survey involving 7,557 residents in Western China reported that participants' overall medication knowledge was “good” (72.77 ± 22.91), whereas their attitudes (32.89 ± 10.64) and practices (71.27 ± 19.09) were rated as “average,” according to established scoring criteria (33). Similarly, a survey of 471 residents in Haikou, Hainan Province, found that participants had “good” overall knowledge levels (52.2 ± 13.08) but only “average” medication-related attitudes (27.34 ± 8.14) and practices (51.54 ± 9.22). These findings highlight an increased risk of irrational medication use, particularly among individuals with lower educational attainment (34). However, a study of 3,272 adult residents in Shanxi Province yielded divergent results, indicating “fair” overall medication knowledge, but “good” attitudes and practices. This study also emphasized higher medication risks among male respondents and individuals from low-income and lower-education backgrounds (35). Such discrepancies suggest that the relationship and translation among KAP components may exhibit varying patterns influenced by demographic characteristics and regional variations.

Furthermore, our results align with studies conducted among university students in other countries and regions, including Saudi Arabia, Thailand, and Colombia, which similarly identified significant challenges in translating medication-related knowledge into safe practices within this population (36–38). This common global challenge underscores the notion that the knowledge-to-practice gap arises not only from individual factors, such as insufficient personal experience, but is also strongly associated with systemic issues. A lack of sufficient emphasis on safe medication practices within educational frameworks and healthcare systems at national and regional levels appears to be a critical contributing factor (39–42). Therefore, the subsequent sections of this paper will draw upon international experiences with interventions to propose targeted strategies designed to effectively bridge this gap and promote the translation of medication knowledge into consistently safe practices among university students.

### 4.2 Analysis of factors influencing KAP

#### 4.2.1 Knowledge dimension

Our findings indicated that students aged 18–20, 21–24, and 25–28 years scored significantly higher in medication knowledge compared to the reference group aged 16–17 years. This suggests that older students may acquire richer medication-related knowledge through increased educational exposure and life experience. However, no statistically significant difference was observed between students over 28 years old and the 16–17-year-old reference group, possibly due to an insufficient sample size. This finding contrasts with an Indonesian community-based survey (43), which reported a significant correlation between age and self-medication knowledge. This discrepancy may stem from population heterogeneity: our study focused on university students within a concentrated age range and standardized higher education setting, potentially minimizing knowledge disparities related to professional background. In contrast, the Indonesian study encompassed a broader age range (18–65+ years), exhibiting greater diversity in life experiences and educational backgrounds, thus making age-related differences in knowledge more pronounced. Therefore, future studies should consider larger sample sizes or more refined measurement tools to thoroughly investigate the impact of age on medication knowledge.

Regarding educational stage, undergraduate and master's students demonstrated significantly higher medication knowledge scores than college diploma students, which aligns with findings reported in previous studies. For instance, a study of Italian university students indicated that higher education levels correlate with greater knowledge of antibiotics (44). Another survey of US college students found that juniors had approximately 1.93 times higher correct antibiotic knowledge rates than freshmen (*p* < 0.05), although the benefit diminished with further progression in grade level, highlighting the importance of introducing antibiotic education early in higher education curricula (45). Moreover, higher education typically includes training in critical thinking, enabling individuals to approach health-related information more critically and cautiously. Studies have shown that students trained in critical thinking acquire, analyze, and apply health information more effectively, resulting in superior medication knowledge and more rational medication use (46, 47). Additionally, students at advanced educational stages typically have greater access to academic resources, facilitating the acquisition of accurate medication information (48, 49). Nevertheless, no significant difference in knowledge scores was observed between doctoral and college diploma students, possibly attributable to the limited number of doctoral participants restricting statistical power.

Part-time enrolled students demonstrated relatively lower medication knowledge levels, indicating potential limitations in the dissemination of knowledge within their educational framework. This may be attributed to the fragmented learning patterns common among part-time students, who frequently balance academic studies with employment responsibilities (50, 51), thus resulting in fewer opportunities for participation in campus-based health-related activities (52, 53). Consequently, this may hinder the systematic development of comprehensive medication-related cognition. Previous research has confirmed that insufficient health literacy significantly increases the risk of medication errors and irrational drug use (54).

Furthermore, the variety of knowledge dissemination channels significantly influenced medication knowledge scores. Students who primarily obtained knowledge from peers and friends exhibited significantly higher knowledge scores, potentially due to the contextualized and interactive nature of peer-based information exchange (55, 56). Peer-to-peer knowledge sharing, frequently grounded in strong interpersonal trust, facilitates easier acceptance and internalization of knowledge. Similarly, health education studies among community diabetes patients have shown that peer-led interventions significantly enhance health knowledge (57). Nevertheless, this informal dissemination method carries potential risks of information inaccuracies, necessitating further research to assess its long-term effectiveness and reliability (58, 59).

#### 4.2.2 Attitude dimension

This study found that university students generally exhibited cautious attitudes toward medication use, a finding consistent with previous research conducted in Brunei, Portugal, Ethiopia, and Pakistan (60–63). This consistency suggests a certain degree of cross-cultural commonality in attitudes toward medication safety among university students.

However, no significant correlation was observed between household monthly income per capita and attitudes toward medication. This finding diverges from previous studies indicating that economic advantage often correlates with higher health literacy (64–68). A possible explanation could be that, as all participants in this study were university students, their attitudes toward medication may have been more substantially shaped by formal education, health-promotion initiatives, and the extensive dissemination of health information through social media, thereby reducing the influence of economic factors.

Concurrently, unique social policies in China may have played a significant moderating role. In recent years, the expansion and enhancement of China's basic medical insurance policies, coupled with significant progress toward achieving universal health coverage, have contributed to substantially reducing out-of-pocket medical expenses for the majority of the population. This has effectively alleviated financial burdens for lower-income families, enabling them to prioritize professional guidance when making healthcare and medication-related decisions rather than basing their choices solely on cost considerations (69–73). Therefore, future strategies for medication safety education should adopt a multifaceted approach, carefully taking into account local social policies and cultural contexts to implement targeted interventions and effectively enhance rational medication awareness among university students.

#### 4.2.3 Practices dimension

This study found that students aged 18–20 and 21–24 exhibited more appropriate and effective medication practices compared with the reference group aged 16–17 years. This finding suggests that increased age may correlate with greater practical experience and improved capacity to translate knowledge into practice. However, no significant improvement in standardized medication practices was observed among students older than 25 years, possibly due to the smaller sample size in this age group or the stabilization of their medication-related behaviors. Existing research indicates that university students undergoing medical or related training do not always demonstrate better translation of knowledge into behavior (74, 75), suggesting that multiple factors influence this process. Future studies should employ more detailed factor analyses and collect longitudinal data with greater representativeness to clarify these findings.

In addition, students who primarily obtained medication knowledge from peers and friends demonstrated more appropriate and effective medication practices. This may be attributed to the interactive and contextualized nature of peer communication, which can reinforce the practical application of knowledge more effectively. Conversely, students enrolled in part-time training programs showed relatively poorer performance in practicing appropriate medication behaviors. This suggests that fragmented educational models may fail to offer adequately continuous and systematic training in medication practices. Furthermore, studies indicate that relying solely on short-term or single-modality health education is typically insufficient for effectively translating knowledge into behavioral practice. The internalization of behaviors and habit formation require long-term, systematic, and interactive educational approaches (76–79). Additionally, a notable mismatch between cognition and behavior may exist, which can often be effectively addressed through repeated and sustained practice, alongside other supportive educational interventions. Consequently, traditional lecture-based or purely knowledge-based teaching methods inadequately address students' decision-making needs when confronted with complex real-world medication scenarios (80, 81). Thus, developing more systematic and interactive intervention frameworks is urgently needed to effectively promote the translation of medication knowledge into appropriate practices among university students.

### 4.3 The “knowledge-practice gap” in public health

The observed disconnect between knowledge and practice in university students' self-medication is not an isolated phenomenon but exemplifies the pervasive “knowledge-practice gap” within public health. Simply enhancing health knowledge does not automatically lead to corresponding improvements in health behaviors, a persistent challenge across various domains of public health, including self-medication.

This gap is especially pronounced in the context of self-medication. For instance, a survey of pharmacy students in Uganda found that while 87.38% possessed good self-medication knowledge and 96.03% held positive attitudes toward medication, only 27.34% actually practiced appropriate medication use, highlighting a significant “knowledge-practice gap” (82). Similarly, a study by Ali et al. involving 1,630 adults in Egypt reported that although 55.0% of respondents had good medication knowledge, this knowledge level did not significantly predict actual medication practices (aOR = 1.15, 95% CI: 0.90–1.48, *p* = 0.268). In contrast, medication attitudes significantly influenced behavior (aOR = 0.44, 95% CI: 0.36–0.55, *p* < 0.001) (83). These findings indicate that individuals, despite being fully aware of the risks associated with self-medication, may still engage in inappropriate practices due to cognitive biases, limited access to healthcare, economic constraints, or other practical barriers.

Similar phenomena are widespread across other areas of public health. Yeh et al. in their study on chronic disease management, found that diabetic patients exhibited high levels of foot care knowledge (86.2% correct responses); however, the correlation between knowledge and actual foot care practices was only moderate (*r* = 0.31), indicating substantial barriers to effectively translating knowledge into behavior (84). Additionally, an Australian study of adolescents aged 15–25 revealed a significant gap between their awareness of healthy eating and physical activity and their actual adherence to related public health recommendations (85). Similarly, a multinational survey by Eltewacy et al. involving 12,606 university students' blood donation behaviors, further corroborated this phenomenon: although health science students possessed significantly higher blood donation knowledge than non-health science students (34.7 vs. 15.7%, *p* < 0.001), actual blood donation rates did not differ significantly between the groups. This emphasizes that actual health behaviors are strongly influenced by factors beyond knowledge alone, including attitudes, healthcare access, and risk perceptions (86).

### 4.4 Recommendations for intervention strategies to improve university students' self-medication KAP

Based on key findings from this study and relevant public health literature, targeted intervention strategies are recommended to comprehensively enhance the appropriateness of university students' self-medication across KAP dimensions.

#### 4.4.1 Knowledge-based intervention strategies

To address knowledge gaps among part-time students, we recommend developing digital micro-learning platforms to supplement their insufficient systematic health education (87, 88). Given the demonstrated efficacy of peer education in facilitating knowledge dissemination and translation, as validated through successful implementations in countries such as the United States and Canada, universities are encouraged to actively implement standardized peer education programs. These programs should incorporate interactive approaches such as classroom discussions, group learning, and social media campaigns. To ensure scientific accuracy, healthcare professionals or research institutions should provide systematic training and content review for peer educators (89–91). Additionally, we advise establishing a collaborative general education framework involving universities, communities, and healthcare systems. Integrating rational medication use into core curricula would promote equitable resource allocation and knowledge dissemination (92).

#### 4.4.2 Attitudinal and policy intervention strategies

To enhance students' cautious and rational attitudes toward medication, universities should consider establishing permanent medication safety consultation platforms. These platforms could leverage online interactions, campus-wide educational campaigns, and pharmaceutical services to reinforce positive attitudes and translate these attitudes effectively into practice. Such integrated intervention models, which concurrently emphasize attitude reinforcement and behavioral guidance, have been shown to be effective and scalable within university settings in New Zealand and the United States (93–95). Concurrently, policymakers should optimize the promotion and implementation of medical insurance policies to alleviate the financial burdens experienced by economically disadvantaged student groups.

#### 4.4.3 Practice-oriented intervention strategies

Considering the limited effectiveness of short-term interventions in modifying entrenched medication behaviors among university students, we recommend integrating core safe medication concepts early into childhood and adolescent educational programs to establish foundational awareness. Research has demonstrated that structured early education significantly improves medication safety awareness and long-term practices (96–99). Furthermore, employing simulation-based learning and scenario-based case studies can effectively assist students in contextualizing and applying theoretical knowledge (100–102). Studies conducted in India, Türkiye, and the United States have consistently shown that sustained medication education, particularly when coupled with pharmacist-led consultations, significantly enhances medication behaviors and overall health outcomes (103–106). To further advance these initiatives, integrating intelligent monitoring systems and real-time big data feedback mechanisms can provide immediate decision-making support. Such an approach not only improves medication decisions in complex scenarios but also effectively reduces polypharmacy risks and medication-related hazards (107, 108). Nevertheless, practical implementation of these systems could encounter significant challenges, including ethical considerations, data privacy concerns, and resource limitations. Therefore, multidisciplinary collaboration and robust policy support are essential for addressing these barriers. Furthermore, additional empirical validation specifically tailored to university student populations remains necessary to confirm the efficacy of these intervention approaches.

### 4.5 Limitations and future research directions

This study has several limitations. First, the cross-sectional design inherently limits the control over potential confounding variables. Thus, unmeasured factors may influence the results, complicating their interpretation. Additionally, the online survey method used in this study, while ensuring participant anonymity and convenience, introduces risks of selection bias and response inaccuracies. Such risks include potential underrepresentation of specific populations and the possibility of careless or rushed responses, all of which may compromise data reliability. Furthermore, data collected exclusively via self-report are susceptible to subjective biases, such as socially desirable responding or idealized self-representation by participants.

Second, the study's limited geographical scope and focus on specific universities and academic majors may restrict sample representativeness. Thus, caution is required when generalizing the findings to broader populations or other regions.

In terms of statistical analyses, the relatively low coefficient of determination (*R*^2^) obtained from multiple regression models indicates that these models accounted for only a modest proportion of the variance in KAP dimension scores. This suggests that other influential variables not included in the current analysis might exist, potentially restricting the comprehensiveness and generalizability of the conclusions. Moreover, the measurement instruments demonstrated limited sensitivity, particularly evident within the medication knowledge dimension, where a high pass rate of 93.50% suggested a pronounced “ceiling effect” (109). This ceiling effect implies that high overall knowledge levels could obscure subtle but meaningful differences among variables, making statistical detection challenging.

Therefore, future research should broaden the geographical and demographic scope of the survey, ensure more representative sampling methods, and integrate objective measurement techniques to enhance validation and extend the current findings.

## 5 Conclusion

This study investigated the characteristics of KAP regarding self-medication among university students in Guangdong Province, China, addressing a gap in the existing literature specific to this region. The findings reveal an imbalance among knowledge, attitudes, and practices in this population. Factors such as age, current academic stage, study mode, and the primary source of medication knowledge were significantly associated with self-medication behaviors.

These results could inform the development of targeted educational interventions and safety-promotion strategies to enhance rational self-medication practices among university students. Future research should investigate specific mechanisms to improve medication behaviors, particularly by evaluating the effectiveness of early-stage interventions, context-specific interventions, and technology-based strategies.

## Data Availability

The datasets presented in this article are not readily available because requests for additional analyses require approval from the Institutional Review Board of the Third Affiliated Hospital of Southern Medical University (Approval no.: 2025-ER-008). Requests to access the datasets should be directed to the corresponding author.
