# Supplementary material for: Self-medication among university students in Guangdong province, China: a cross-sectional study using the KAP model
Source: Front Public Health. 2025 Jul 14;13:1601731. doi: 10.3389/fpubh.2025.1601731 (PMC12301323; doi:10.3389/fpubh.2025.1601731)
Supplement: Supplementary file 1 [file Table_1.docx]

Survey on Knowledge, Attitudes, and Practices (KAP) of Self-Medication Among University Students in Guangdong Province

Section 1

Basic Information

1. Gender

□ Male

□ Female

2. Age

□ 16–17

□ 18–20

□ 21–24

□ 25–28

□ Over 28

3. Current academic stage

□ College diploma student

□ Undergraduate student

□ Master’s student

□ Doctoral student

4. Major

□ Medical/Pharmaceutical-related

□ Non-medical/pharmaceutical

5. Study mode

□ Full-time

□ Part-time

6. School type

□ Double First-Class university

□ Non-Double First-Class university

□ Vocational college

7. Location of your School

□ Central urban area

□ Non-central urban area

8. Family residence

□ Rural

□ Urban

9. Monthly household income per capita (CNY)

□ Below ¥1,000

□ ¥1,000–3,000

□ ¥3,000–5,000

□ ¥5,000–8,000

□ Above ¥8,000

10. Primary source of medication knowledge

□ Family

□ Internet

□ School

□ Friends/peers

□ Books, newspapers, magazines

Section 2

The following statements reflect opinions and perceptions about medication and its use. Please rate your agreement using a scale of 1 (Strongly Disagree) to 5 (Strongly Agree).

1. Getting injections or IV drips when sick ensures faster recovery.

2. Injectable medications (e.g., injections, IV drips) are safer than oral medications.

3. More expensive medications are safer.

4. More expensive medications are more effective.

5. Combining multiple medications improves efficacy.

6. Prolonged medication use leads to better outcomes.

7. Medication should be taken immediately when feeling slightly unwell.

8. Health supplements are equivalent to medications.

9. Taking health supplements reduces the required dosage of medications.

10. Oral medications can be taken with milk, coffee, or beverages.

11. Health supplements can be taken alongside medications.

12. Adjusting IV drip speed during hospitalization is acceptable to shorten treatment time.

13. Antibiotics can cure any cold or fever.

14. Antibiotics can kill all bacteria and viruses.

15. Avoiding antibiotic misuse prevents drug resistance.

16. Using multiple antibiotics speeds up recovery.

17. Antibiotics are anti-inflammatory drugs.

18. Over-the-counter (OTC) drugs have no side effects.

19. Unused medications should be stored in a refrigerator.

20. Stopping medication is acceptable if symptoms improve.

21. Reducing dosage is acceptable if symptoms improve.

22. Increasing dosage is acceptable if symptoms worsen.

23. Switching medications is acceptable if symptoms worsen.

24. New drugs are safer and more effective than older ones.

25. Taking antibiotics to prevent illness when others are sick.

26. Judging medication quality by brand reputation.

27. All medications can be stored at room temperature.

28. Prioritizing efficacy over cost when purchasing medications.

29. Self-medicating based on personal experience or advertisements.

30. Requesting specific medications from doctors during consultations.

31. Trusting pharmacists’ recommendations when purchasing medications.

32. Purchasing prescription drugs with a valid prescription.

33. Regularly checking stored medications at home/dorm.

34. Storing medications where children can easily access them.

35. Checking storage conditions on medication labels.

36. Reviewing side effects and their symptoms in medication instructions.

37. Understanding contraindications (e.g., alcohol, driving).

38. Checking expiration dates before taking medications.

39. Verifying approval numbers on medication packaging.

40. Disposing of expired medications in the trash.

41. Forgetting to take medications as prescribed.

42. Taking expired medications.

43. Taking medications with milk, tea, or coffee.

44. Considering personal health conditions when using medications.

45. Increasing dosage without medical advice to enhance efficacy.

46. Ignoring recommended intervals between doses.

47. Switching medications without advice if no improvement.

48. Reducing dosage or stopping medication after symptom relief.

49. Combining Chinese and Western medicines without guidance.

50. Crushing or splitting tablets before ingestion.

51. Mixing multiple medications without professional guidance.

52. Adding medications to speed up recovery.

53. Refusing medication or stopping use due to listed side effects.

54. Attending community lectures on rational medication use.

55. Attending hospital/community health center lectures.

56. Reading community-distributed materials on rational medication use.

57. Consulting pharmacists at community/street events.

58. Reading hospital/community health center materials.

59. Accessing online/WeChat resources on rational medication use.

60. Participating in university lectures/publications on medication safety.

61. Attending community lectures on rational medication use.

62. Attending hospital/community health center lectures.

63. Reading community-distributed materials.

64. Reading hospital/community health center materials.

65. Accessing online/WeChat resources.

66. Participating in university lectures/publications.
